# Restorative Retelling for Processing Psychedelic Experiences: Rationale and Case Study of Complicated Grief

**DOI:** 10.3389/fpsyg.2022.832879

**Published:** 2022-05-03

**Authors:** Débora González, Marc B. Aixalà, Robert A. Neimeyer, Jordi Cantillo, Donald Nicolson, Magi Farré

**Affiliations:** ^1^Fundación BeckleyMed, Barcelona, Spain; ^2^PHI Association, Barcelona, Spain; ^3^International Center for Ethnobotanical Education, Research and Services (ICEERS), Barcelona, Spain; ^4^Portland Institute for Loss and Transition, Portland, OR, United States; ^5^Department of Psychotherapy, Bath Spa University, Bath, United Kingdom; ^6^Clinical Pharmacology Unit, Hospital Universitari Germans Trias i Pujol and Institut de Recerca Germans Trias i Pujol (IGTP), Barcelona, Spain; ^7^Department of Pharmacology, Therapeutics and Toxicology, Universitat Autònoma de Barcelona, Barcelona, Spain

**Keywords:** ayahuasca, grief, restorative retelling, integration, psychedelic therapy

## Abstract

**Rationale:**

Many psychedelic experiences are meaningful, but ineffable. Engaging in meaning-making regarding emerging symbolic content and changing previous schemas have been proposed as mechanisms of change in psychedelic therapy.

**Objective:**

Firstly, we suggest the implementation of a Restorative Retelling (RR) technique to process and integrate the psychedelic experience into autobiographical memory, in a way that fosters meaning-making. We also show how ayahuasca has the potential to evoke key psychological content in survivors, during the process of grief adjustment following the death of a loved one.

**Methods:**

The rationale for the implementation of RR to process psychedelic experiences and a case study of a woman suffering from Complicated Grief (CG) after her mother’s suicide are presented.

**Results:**

Evaluations conducted before the ayahuasca experience and after RR suggest the effectiveness of ayahuasca and RR in reducing symptoms of CG and psychopathology.

**Conclusion:**

This case report illustrates an effective adaptation of the RR technique for processing the psychedelic experience. The significance of the study and its limitations are discussed.

## Introduction

Ayahuasca is an Amazonian brew used as traditional medicine by more than 70 different indigenous communities (Schultes and Hofmann, 1992), and as a sacrament by several religious groups including Santo Daime, União do Vegetal (UDV), and Barquinha (Labate and Araujo, 2004). “Ayahuasca” is a Quechua term derived from the words: *Aya* (dead, soul, or ancestor) and *Huasca* (rope or vine), translatable as “vine of the souls” or “vine of the dead” (Metzner, 2005). Clinical trials provide evidence that ayahuasca could be effective for treating depression (Osório et al., 2015; Palhano-Fontes et al., 2019). Preliminary scientific results also show ayahuasca’s potential in treating addiction (Thomas et al., 2013) and grief following the death of a loved one (González et al., 2019, 2020).

The main components of ayahuasca are *N,N*-Dimethyltryptamine (DMT), present in the shrub *Psychotria viridis*, and the alkaloids harmala, harmine, and harmaline, found in the vine *Banisteriopsis caapi* (Schultes and Hofmann, 1992). Ayahuasca’s effects are dose-dependent (Riba et al., 2003) and the nature of an individual’s psychedelic experience is determined by their mindset as well as by physical and cultural environmental influences (Hartogsohn, 2017). Effects begin around 30–40 min after oral intake and last up to 4 h. Psychological effects include changes in spatio-temporal perception, sensorial alterations, synesthesia, increased emotional arousal and emotional lability, increased introspection, reflections, biographical memories, associative thinking, insights, and changes in the sense of “self” (Riba et al., 2001, 2003). Particularly striking are the visions that occur mainly with closed eyes, which encompass a rich panorama ranging from geometric patterns to supernatural imagery and long conversations with an imagined “other” (Shanon, 2002, 2003). However, as under the effects of other psychedelics, participants may find it difficult to express their experience in words, both during the effects and *a posteriori* (Preller and Vollenweider, 2016). However, the ineffability of such experiences is not incompatible with their remarkable tendency to enhance perception of meaning (Winkelman, 2017; Hartogsohn, 2018).

One psychotherapeutic model attracting growing attention for treating grief from the death of a loved one is the constructivist approach, which views grieving as a process of reconstructing a world of meaning that has been challenged by loss (Neimeyer, 2015, 2019). Several studies have shown that meaning-making is a predictor of adaptative bereavement (Currier et al., 2006) and a potential mediator of bereavement adjustment (Milman et al., 2017, 2019). Given psychedelics’ ability to enhance perception of meaning and that several qualitative studies have shown that ayahuasca naturally evokes new meaningful experiences related to the grief process in bereaved individuals (González et al., 2019, 2021), the process of “re-constructing a world of meaning” promoted by constructivist psychotherapy could be significantly enriched by new information that emerges during psychedelic experiences. Furthermore, since psychedelics act as non-specific amplifiers of psychological material (Grof, 1994), the techniques employed in psychotherapy may facilitate an appropriate mindset for the emergence of psychological content related to the grieving process during psychedelic experiences. In this way, the benefits derived from constructivist psychotherapy and ayahuasca ceremonies could complement and enhance one another within a framework of psychedelic-assisted psychotherapy. This therapeutic model favors the use of one or a few high doses of psychedelics to create an overwhelming and transcendent experience to catalyze the therapeutic process (Garcia-Romeu and Richards, 2018). The combination of drug administration embedded within talk therapy aims to facilitate processing the psychedelic experience and potentiate novel insights into the patient’s condition (Pahnke et al., 1970).

### The Rationale for Restorative Retelling for Processing Psychedelic Experiences

Narrative is the way by which and in which everyday experience is processed (Wigren, 1994). Encoding experience through language allows it to be integrated into semantic and episodic memory, facilitating retrieval and reflection (Irish and Piguet, 2013). The culturally shaped cognitive and linguistic processes that guide the self-telling of experiences allow us to structure perceptual experience, organize memory, and purpose-build the very “events” of an experience (Bruner, 1987). However, it is often challenging to craft a narrative about a psychedelic experience. Psychedelics modify neural hierarchies and the flow of information, reducing top-down control, and enhancing bottom-up information transfer (Carhart-Harris et al., 2012; Alonso et al., 2015). This allows information from sensory, emotional, and memory areas to emerge into consciousness without the constraints normally exerted by the prefrontal cortex based on previous knowledge and expectations. In addition, decreased activation of areas involved in language processing such as Broca’s area (BA 44) has been observed during ayahuasca ingestion, while subjects were performing a classical verbal fluency task (Prado et al., 2009). All this is reflected in the access to a deeply internal world that is not easy to process semantically.

Similar to psychedelic experiences, trauma victims are often unable to form narratives of traumatic experiences (Wigren, 1994). On a neurocognitive level, traumatic experiences involve decreased activity in Broca’s language-processing area and increased activity in areas that govern intense emotions and visual images, such as the amygdala and the right secondary visual cortex (Rauch et al., 1998). This leads to incoherence and disorganization of trauma narratives that correlate with trauma symptoms (Foa et al., 1995).

Schemas and concepts are central to the constructivist approach. A schema is a cognitive structure containing a concept and the relationships among its various attributes (Fiske, 2004). At a cognitive level, traumatic experiences are too alien and too discrepant from previous schemas to be automatically integrated (Wigren, 1994). Unexpected experiences that do not fit into previous existing mental schemas can become dissociated or forgotten. The creation and evolution of schemas occur through the dual processes of *assimilation* and *accommodation* (Furth, 1969). In the former, familiar experiences are categorized and incorporated into existing schemas, thereby strengthening them; in the latter, schemas are modified in order to account for novel experiences that cannot be categorized into existing schemas.

Lastly, as may happen with some psychedelic experiences, people affected by certain traumatic events may perceive the experience as too intimate to be told, fear the implications it may have on others, or fear being misunderstood or socially censured. On this relational level, traumatic experiences are typically only related as a highly “edited” or censored story to others, often resulting in a “silent story” that lives continuously in the traumatized person’s own rumination (Neimeyer, 2019).

For these reasons, the constructivist model of grief intervention (Neimeyer, 1999, 2019, 2022; Gilbert, 2002; Shear et al., 2011) identifies narrative reconstructions of the trauma experience as central to the therapeutic process. This model involves integration of sensory trauma memories by representing them linguistically as part of the healing process within the trauma narrative (Wigren, 1994; Peri and Gofman, 2014). In this narrative technique, traumatic experiences are assimilated and accommodated into previous schemas using questions like: *How do my religious or philosophical beliefs help me accommodate this experience* and *how are they changed by it in turn?* (Neimeyer and Thompson, 2014).

Based on the aforementioned similarities between traumatic and psychedelic experiences and the long-term benefits of meaning-making through narration, we suggest an adaptation and implementation of Restorative Retelling (RR) for semantically processing psychedelic experiences, including but not restricted to those related to a grief process. The adjustment of RR for the processing of ayahuasca experiences is described here through a detailed evidence-based case report.

### Restorative Retelling

Restorative Retelling is a clinical procedure in which a trained mental health professional supports a bereaved client in closely reviewing and relating the story of a traumatic event, or in this case a psychedelic experience, under conditions of high safety and low avoidance (Rynearson, 2006; Salloum and Rynearson 2006; Neimeyer, 2012a, 2019; Neimeyer and Rynearson, 2022). Sometimes drawing is encouraged to externalize and illustrate any “hot spots” and facilitate their integration (Correa, 2016).

a. The **assimilation process** involves the following steps: First, grounding the client in personal, relational, or community sources of resilience. Second, inviting the client to vividly relive the experience, using the present tense to potentiate emotional engagement with the memory. Third, slowly panning the “camera” of attention over the details of each scene, noting emotionally significant material. Fourth, moving naturally among different narrative voices:
  - The *external narrative* is the account of what happened during experience. The key is to listen carefully, facilitating the development of a detailed sequential narrative.
  - The *internal narrative* is the story of what was happening inside the client as critical aspects of the experience were unfolding around them. The key is to allow clients to acknowledge the intimate impact of their story.
  - The *reflexive narrative* is the meaning-oriented story, which is often suggested by the therapist’s implicit questions and the client’s interpretations of the events being related. The goal is to trace and create space for the client to make fuller sense of the psychedelic experience.

The final step involves repeatedly reviewing any “hot spots” by regulating the upsurge of strong emotions through mindful breathing or visual distancing from the scene. Emotional processing is facilitated when the client is fully engaged with the memory but at the same time is grounded in the present and not emotionally overwhelmed (See Neimeyer, 2012b and Neimeyer and Rynearson, 2022 for a broader review).

The process of assimilating the experience may take several sessions. When this occurs, the narrative should be punctuated in closed chapters that allow the client to go home feeling safe, avoiding rumination about the experience. Importantly, sufficient time should be allowed for processing the experience, where the therapist and client discuss any insights that the client may have gained from it.

b. The **accommodation process** focusses on a reflective assessment and reorganization of the internal world. Our inner world can be understood in terms of schemas that act as a cognitive-emotional guide for perceiving ourselves, people, and events, and effectively planning and acting in our world (Epstein, 1990). Given that the assimilation of psychedelic experiences has a powerful capacity for mediating major shifts in perspective (Forstmann et al., 2020; Timmermann et al., 2021), the goal of this procedure is to prompt a psychological increase, expansion, or development in cognitive-emotional understandings of themselves, others, and the world.

Useful questions to evoke a reflective assessment of the patient’s belief system are as: *Who am I in light of this experience? How does it fit into my existential sense of how the world operates?* (based on Neimeyer and Thompson, 2014). In psychedelic therapy, it can be useful to redirect questions to any specific issues on which clients feel blocked during a psychotherapy process. Special attention must be taken to nurture the reflective process until the client formulates an adaptive meaning-making of their belief system. Three routes have been described to foster posttraumatic growth: strength through suffering, including self-discovery and new self-perception produced over the course of coping and adaptation; the creation of value triggered by a perception of human fragility; and greater complexity and structural growth (Janoff-Bulman, 2014).

### Objective

In this paper, we present a case study describing the adaptation of Restorative Retelling for the integration of an ayahuasca experience with a client suffering from complicated grief (CG). The client drank ayahuasca as part of a pilot study for the design of a psychotherapeutic intervention to prevent prolonged grief disorder. We show the changes following the intervention, as measured before the ayahuasca experience and after the Restorative Retelling procedure. We hypothesized that the combined effects of the ayahuasca experience and the Restorative Retelling procedure would lead to an improvement in CG symptoms. The ayahuasca experience narratives were recorded and are shown here in the form of vignettes. The description of the process has a 2-fold objective: to exemplify the use of the Restorative Retelling technique and to show how ayahuasca can naturally evoke key psychological content in the process of grief adjustment.

## Materials and Methods

### Setting

Ayahuasca sessions were conducted in groups of seven participants supported by four facilitators in a private clinic in Barcelona. Participants were allowed to sit or lie on mattresses while listening to a playlist of selected music while blindfolded with a mask.

### The Participant

A.O. was a 29-year-old queer woman and the elder of two children. A.O’s mother completed suicide at home by throwing herself off the balcony, leaving A.O. and her younger brother alone in their house aged 4 and 2, respectively. However, A.O was not present in the room when her mother died by suicide and has hardly any memory of her. A.O. lived her whole life believing the story her family told her about how her mother died in an accident. At the time, she gave her consent to participate in the pilot study, A.O’s aunt had confessed to her the truth about her mother’s suicide story. These provoked overwhelming feelings of grief and rejection toward her family for having felt cheated throughout her life, especially by her father. She also complained of having trouble moving forward with her life due to an intense longing for her mother and a family she could trust. She scored 51 on the Inventory of Complicated Grief (ICG) at the baseline assessment, meeting the criteria for complicated grief (Prigerson et al., 1995).

### Procedure

The course of the pilot study involved 14 weekly psychotherapeutic sessions and three ayahuasca experiences. The ayahuasca sessions were carried out in groups of four participants supported by five facilitators. Consistent with Greer and Tolbert (1998), facilitators created a setting of safety and support trying to limit their interventions to the explicit requirements of the patient. The sessions were accompanied by a curated soundtrack with alternating periods of silence. Participants followed a schedule, meeting at the clinic at 9 am. After explaining the program of the session, participants’ weights were measured in order to calculate the exact dose for each one. At 10 am, different techniques were carried out to encourage communication among participants and to anchor the topic of grief in their mindsets (Hedtke, 2012; Rollo-Carlson, 2015; Chow and Chu, 2016). At 11 am, the ayahuasca session began, lasting 4 h. After the ayahuasca session, participants were offered a vegetarian meal. At 4 pm, art materials were offered so that participants could express their experience on a visual artistic level. At 5 pm, a sharing circle was held in which participants could share their experience with the other members of the group. During this dynamic, participants were encouraged to cultivate active listening and not give any type of feedback on the experiences of their peers. At 7 pm, the specific questionnaires were distributed to assess the psychological state of each participant. The sessions of retelling the narrative of the ayahuasca experience were conducted individually by the psychologist in the same clinic from 3 to 5 days after the ayahuasca experience. Each narrative reconstruction session lasted approximately 90 min.

The psychological content that emerged during the first ayahuasca session in this case study was not directly related to the grief process. Immediately following the second ayahuasca session, A.O. unexpectedly relived the traumatic event of her mother’s suicide, visualizing details of the scene she never witnessed. The Restorative Retelling of the second experience brought some posttraumatic growth, *via* the route of “strength through suffering.” The narrative of the third ayahuasca experience is described in Qualitative Outcomes.

### Measures

#### Inventory of Complicated Grief

The ICG (Prigerson et al., 1995) is a self-report questionnaire that assesses indicators of pathological grief in 19 Likert-type items scored from 0 to 4. Higher scores imply higher severity of grief, with a score of ≥30 indicating complicated grief (Shear et al., 2005).

#### The Symptom Check-List-90-Revised

The Symptom Check-List-90-Revised (SCL-90-R; Derogatis, 1994) is a self-report questionnaire that uses 90 Likert-type items scored from 0 to 4 to assess nine psychopathological symptomatic dimensions: Somatization (SOM), Obsessive–Compulsive (O–C), Interpersonal Sensitivity (I–S), Depression (DEP), Anxiety (ANX), Hostility (HOS), Phobic Anxiety (PHOB), Paranoid Ideation (PAR), and Psychoticism (PSY). In each scale, higher scores imply worse symptomatology.

### Ayahuasca Analyses and Dose

The ayahuasca was provided by the Santo Daime church, Estrela D’Alva (Barcelona). The ayahuasca was prepared by boiling the stems of *Banisteriopsis caapi* and *Psychotria viridis*. Analyses were carried out by Energy Control^1^ using liquid chromatography-mass spectrometry (LC–MS). Ayahuasca contained 0.5 mg/ml DMT, 0.03 mg/ml tetrahydroharmine, 0.08 mg/ml harmaline, and 1.4 mg/ml harmine.

Two doses were administered in each ayahuasca session: a first standard dose for all participants of 40 ml and, 35 min later, a second dose of 97 ml in the case of this participant (2 ml/kg body weight). Measurement of the second dose was consistent with previous literature, which establishes 1.0 mg DMT/kg body weight as a high ayahuasca dose (Riba et al., 2001). This dosing schedule was based on the estimated metabolization time for monoamine oxidase inhibitors (MAOIs) to achieve second-dose complete absorption of DMT (Riba et al., 2003).

### Statistical Analyses

The Reliable Change Index (RCI) for ICG and SCL-90-R was computed according to Jacobson and Truax (1991) and Bauer et al. (2004), using previous data sets to obtain the SD and a coefficient for each measure (Prigerson et al., 1995; Sánchez et al., 2002; Tomioka et al., 2008; Parro-Jiménez et al., 2021). In this study, values of *p* < 0.05 were considered statistically significant. Statistical analysis was performed using Statistical Package for the Social Sciences (SPSS for Windows, version 20).

## Results

### Qualitative Outcomes: Restorative Retelling of the Third Ayahuasca Session

#### Assimilation

Given that a therapeutic alliance already existed between A.O. and the therapist, the session began directly with body-awareness and relaxation exercises. Next, A.O. was invited to close her eyes and remember what happened during the ayahuasca ceremony. She was asked to identify the most emotionally charged fragments of her experience and organize them into a coherent narrative. When she felt ready, she began to retell the experience in detail, dividing it into two parts.

##### First Part

A.O. describes how she went “*to another place, suddenly with a jolt*” and began to have “*loads of visions: of animals, shapes, colors, junk*,” with feelings of a “*panic I’d never felt before, like in a dream*.” She was afraid of “*losing control*” and “*being alone*.” She felt that in this other place “*nobody could help me*.”

*Participant: They were like a figure, one on top of the other… they turned into a totem pole, the totem was a mask, with like, aggressive faces… like sneering demons sticking out their tongues, long tongues. They were in front of me, they came for me and grabbed at me… it was like… fuck! Where the hell has this come from? It’s so real!… This actually exists in some place, and I’m in that place…*

*Therapist: Do these figures mean anything to you?*

*Participant: The figures? (shakes her head) …no… well, yeah, they do kinda but I would not know how to describe it.*

*Therapist: What do these figures suggest to you?*

*Participant: I do not know if I’ve been conditioned from seeing Mexican art… but now that I think about it, it was like they were guardians, entities, and spirits.*

*Therapist: Why do you think they appeared at that moment? What role could they be playing?*

*Participant: … they were there to make me understand… or to make me aware of other states. They appeared in order to show themselves. So that I could “see.”*

In this vignette, the therapist intervenes in the narrative, evoking a reflective process about the meaning of the images that the participant considers to be “real.” The result was the construction of positive meaning around a phenomenon that did not previously make sense and produced feelings of panic during the experience.

A.O. continues describing her experience: “*In all that mad situation, since I saw that no one on earth could help me, I asked for my mother*.”

##### Second Part

###### Mother and God

Next, the participant describes how, as she was laying on her mattress during the session, she felt her mother sitting on the edge of the mattress beside her and began to feel in a “*more defined place, with an aura that was, like… heavenly! Like, transparent, as if I was surrounded by a kind of fabric, like a sheen… and this gave me a lot of calm*.”

*Participant: And I remember that there was also a guide, like an entity or something … that gave me the feeling of this… ‘Everything’, you know?*

*Therapist: this ‘Everything’?*

*Participant: Well, I got the feeling that it’s what people call God, you know? A god… like God is Everything. It was an energy that was like, unearthly. And it… this energy brought me to my mum. It had a really strong bond with my mother.*

*Therapist: How do you feel when you are with your mum and this energy that’s ‘like god’?*

*Participant: Well, the way I feel it, it’s like how love is unconditional, but love like… (she hunches over and thinks) like love is the be-all and end-all. It’s the meaning of life.*

After being asked about the internal narrative, the participant recovers memories stored in her episodic memory, and, reflecting on them, she constructs a new metaphysical meaning of reality, with great potential for reorganizing and making sense of her life.

###### Mother and Relatives

The participant describes how her mother went in and out of the scenes, showing different situations to her. In the next scene, mother and daughter are watching a family meal from outside. During this scene, the mother communicates to the participant a need “*to have a kind of alliance with me, admitting that things had been tough (family dynamics after the suicide), that she understood that my relationship with my father was difficult … with my uncles… but that I had to understand it all from their point of view*.”

*Therapist: She wanted you to understand your family?*

*Participant: Yeah, that she’d left behind a really difficult situation for everyone… and that they all had their own crosses to bear, their own stuff to deal with and they could not have dealt with things any differently, same as I could not have… She wanted me to see beyond the surface of things.*

*Therapist: So that dialog with your mother allowed you to understand your relatives from another point of view?*

*Participant: Yeah.*

*Therapist: And what did you feel, being able to understand them differently?*

*Participant: I felt, like, unconditional love. And at the same time I felt so sorry, like, a deep sense of pity… because they had lost a sister, and the way they lost her… and my father was her partner. Perhaps my father is a waste of space, who’s maybe never done anything right, never helped me to get better, but that’s not… not the whole picture.*

By carefully integrating the external and internal narratives of the ayahuasca experience, the participant is able to assimilate this new information about her family and can also accommodate previous schemas that had perpetuated difficult family relationships, in order to adopt a more compassionate attitude.

###### Mother Asks for Forgiveness

In the next scene, the participant and her mother meet each other in private “*and she just broke down… she begged me for forgiveness, and she accepted responsibility for the way things had turned out. It was as if she had not been able to cure herself in life, she was never able to see what I was seeing*….”

After the therapist digs a little into the meaning of this conversation, the participant explains, “*I’ve always had this need to have a talk with my mother that I could never have, and nobody could take her place*.” This fragment clearly demonstrates a potential resolution of “unfinished business.”

###### Mother During the Coma State

A.O. continues explaining the next scene of her experience.

*Participant: Then I’m in another situation where it’s like I’m there, as if I was her, in the hospital bed, and I’m, like, not completely dead yet… I think she must’ve felt so bad in that moment. I do not know if it’s just my projection because it’s something I’ve thought about so many times…*

*Therapist: … what exactly did you experience in that moment?*

*Participant: I was lying on the bed like this (she lies down), and suddenly, I do not know how, I lifted my hands (she crosses her hands across her chest) …and in that moment… bam!… I experienced being in a coma. Then I got hit by this huge wave of sadness, that was the same sorrow that she was going through in that state, my mum. And then I became aware of, like, fuck, I’ve gone and thrown myself off the balcony, I’ve left my two children in the house, this has really happened, I’m here… and so much sorrow, so, so much sorrow (she buries her face in her hands).*

In this vignette, the therapist refocuses the participant’s story, eliciting the external voice of the experience to redirect the narrative toward what happened during the experience, in order to preserve the narrative thread and continue with the assimilation of the experience.

In this case, we see how copying certain body positions that she had while under the effects of ayahuasca helped the participant to evoke clearer memories of her experience.

###### Funeral

A.O. describes how in the following scene she becomes a participant in a new funeral for her mother, since she was not allowed to attend the real funeral.

*Participant: …and the funeral, well it’s happy. I mean, like, not super happy… it’s emotional. As if everything I was experiencing with all this energy (the “Everything” or “God”) was being projected onto all of us, and the family was soaking it all up, you know?…*

*Therapist: What emotions were there?*

*Participant: like, a feeling of community… of being at peace with the process of death. Like, understanding that death has some kind of meaning.*

*Therapist: What meaning does death have, for you?*

*Participant: …that everything’s not like it seems… that there are laws that move things that have a deeper consequence, that we cannot see right now, and maybe we’ll never see in this life. If we could see it in levels, like… this happens here, but also there (she gestures with raised hands), what’s happening is something else. So then you say… ah, right! (death) is just another collateral damage, nothing more. But you only see someone who’s sad, who’s suffering, who leaves her kids in a flat, taking her own life… it’s wild.*

Following the natural course of the narrative, eliciting the reflexive inner voice, the participant constructs a richer and more complex hierarchy of beliefs, which allow her to accommodate her previous schemas regarding death.

#### Accommodation

The accommodation process of the ayahuasca experience centered on the schemas that the participant previously held regarding her mother, her relatives, the world, and herself. Here, we look at the accommodation process regarding the schema of her mother.

*Therapist: Okay…, thank you for sharing your experience. If you wish, you can open your eyes when you are ready (Leaving enough time for her to sit up and open her eyes) …How do you feel?*

*Participant: (smiling) Good.*

*Therapist: Now, let me ask you a few questions about your experience. Do you think that this experience has changed the impression you had about your mother, in any way? Is there any kind of knowledge or information you have now, that you did not have before?*

*Participant: Yes, the only thing is, like… the reality of everyday life makes you… (she sighs) I dunno. But it’s definitely left its mark; something has changed. There’s a bit of it that will not be the same as before, but I do not know if it’s specifically about how I see my mother… Like, after the experience, I get the feeling that I even see her differently in the photos.*

*Therapist: Differently? Can you tell me about those changes in the photos?*

*Participant: It’s still her, but her attitude has changed… she seems more calm, more serene… like she had this dark, heavy energy before… but now it’s like I look at the photo and I remember that second part (of the experience), that language… then, I look at the photo in a different way, I explain it to myself differently, and I see her in a different way.*

*Therapist: And has all this promoted any change in how you relate with her?*

*Participant: Yes, because I think that now she’s… closer, more… accessible! …it used to be so disturbing because she’s someone who disappeared and just… she was gone, there was nothing. Nothing! And now I have my little place (clasping her hands across her chest) where, if I want, I can find her. There’s a space here where we can find each other. Not in the human and material way that I want, but now she’s closer and more within reach. I have more tools and resources, because I’ve seen it and experienced it, so I know that if I want, and if I make the effort—because I see it as an effort—I can access her. I can ask her to be there, and she’ll be there.*

In this case, concrete questions about the experience’s impact on schemas that play a key role in the grief process facilitate reflection and a reconstructed bond of connection with her mother in the present moment.

### Quantitative Outcomes: Assessment

Complicated grief symptoms and psychopathology were assessed before taking ayahuasca (third session) and after the restorative procedure (Table 1). The participant did not meet the criteria for CG in the post-assessment. Depressive symptomatology was also significantly reduced at the post-test (*p* < 0.001).

**Table 1:** ICG and SCL-90-R subscales before ayahuasca ingestion and after Restorative Retelling (RR).

	Score		
	Before	After	RCI
ICG	34.00	7.00	−3.59^***^
SCL-90-R			
Somatization	0.42	0.25	−1.31
Obsessive–compulsive	0.20	0.20	0.00
Interpersonal sensitivity	0.44	0.22	−1.12
Depression	1.38	0.62	−3.88^***^
Anxiety	0.10	0.10	0.00
Hostility	0.33	0.17	−0.58
Phobic anxiety	0.14	0.14	0.00
Paranoid ideation	0.83	0.33	−1.91
Psychoticism	0.60	0.40	−1.20

*** *p* ≤ 0.001.

RCI: Reliable change index was calculated according to Jacobson and Truax (1991) using the formula of Bauer et al. (2004).

## Discussion

### Restorative Retelling for Processing the Ayahuasca Experience

This case report shows an adaptation of Restorative Retelling—an approach originally developed to construct narratives around traumatic death (Rynearson, 2006)—for processing psychedelic experiences. The results provide preliminary support for Restorative Retelling’s effectiveness following psychedelic experiences, showing a clinically significant decrease in psychopathology levels as measured before the ayahuasca experiences and after the retelling process. This improvement in ICG symptoms and GSI was consistent with studies showing a link between organized, coherent narratives and a general decrease in psychopathology (Greenberg and Angus, 2004), PTSD symptoms (Foa et al., 1995; Jelinek et al., 2009), depression (Nelson and Horowitz, 2001), and grief (Barbosa et al., 2014).

Retelling the narrative of an event is a technique which can reduce Prolonged Grief Disorder symptoms (PGD; Boelen et al., 2007; Simon, 2013; Peri et al., 2016). However, our adaptation of this technique includes some novel characteristics. Dividing the process into two sections—one focused on assimilation and the other on accommodation—is not indicated in the original technique, where both are covered naturally during dialog between patient and therapist. In our adaptation of this technique for psychedelic experiences, we have established that the accommodation of schemas also emerges naturally during the assimilation phase, especially when the reflexive narrative is evoked. Nevertheless, given that psychedelic experiences have a powerful capacity for mediating major shifts in perspective (Timmermann et al., 2021), we considered it may be relevant to dedicate a section focused specifically on the accommodation of key schemas in the patient’s therapeutic process. This not only encourages assimilation and meaning-making of content that emerged during the psychedelic experience, but also facilitates the reconstruction of a richer and more complex reality. When changes to previous schemas are particularly profound, we consider it appropriate that patients receive ongoing support from the therapist during their adaptation to this new reality. In this way, the health professional can maximize long-term therapeutic potential and minimize any risks derived from carrying out major changes that could affect third parties.

Changing previous schemas and meaning-making of emerging symbolic content may be mechanisms of change in psychedelic therapy (Meikle et al., 2020; Hearn, 2021; Nayak and Johnson, 2021). Restorative Retelling fosters both processes, facilitating the integration of the experience into autobiographical memory and promoting its long-term benefits. Moreover, it is consistent with the inner-directed approach used in psychedelic therapy, where the therapist encourages the client to look into their inner experience for insights (Mithoefer, 2017; Phelps, 2019). We hope this technique may complement other techniques for psychedelic experience integration such as psychedelic-assisted psychotherapy practices, somatic techniques, and mindfulness based-modalities (Gorman et al., 2021).

### Therapeutic Potential of Ayahuasca With Grief

This case report highlights ayahuasca’s potential for evoking the emergence of key psychological content in the adaptation of grief processes. In this experience with ayahuasca, the participant connects with the presence of her mother, and together they go through a series of different scenarios. This particular experience was evoked by ingesting ayahuasca, but between 50 and 80% of bereaved people experience a “sense of presence” of the deceased person, either in daily life or in vivid dreams, without the use of psychedelics (Steffen and Coyle, 2010). These experiences of a “sense of presence” are no longer considered to be symptoms of an underlying mental health condition nor a sign of pathology (Hayes and Leudar, 2016). Although in some cases, these experiences can cause distress, most bereaved individuals report feeling reduced loneliness, less intense pain from the loss, a helpful sense of connection and comfort, and receiving guidance or encouragement from the deceased (Hayes and Leudar, 2016; Jahn and Spencer-Thomas, 2018). These experiences can help create a framework for meaning-making following the death of a loved one, facilitating increased coping and growth, benefit-finding, and identity-change processes that can lead to posttraumatic growth (Steffen and Coyle, 2010).

We want to emphasize that, from our perspective, the most relevant aspect in our participant’s adaptation of grief was a significant shift in her schema or “internal working model” of her mother, facilitated by the experience of “contact” with the presence of her mother. In this case, a new image of her mother, serene and calm, replaced the dark and tragic representation of her that the participant had previously harbored. Schemas or “internal working models” provide a connection with attachment figures, even in their physical absence, and the quality of this schema determines the attachment style with the loved one (Bowlby, 1982; Bartholomew and Horowitz, 1991). This implies that the transformation in the participant’s internal representation of her mother has the potential to transform the style of bond she has with her, probably moving from an insecure or anxious style to a secure bond. The new paradigm in the adaptation of grief centers on continuing the bond with the deceased after death, rather than detachment and “moving on” in a linear way (Klass et al., 2014). For this reason, fostering a secure attachment with the deceased is the objective of various contemporary techniques employed in grief therapy (Neimeyer, 2012b, 2016, 2022; Kosminsky and Jordan, 2016). This is because secure relationships with attachment figures facilitate biological regulatory responses such as affective, attentional, and motivational processes (Shear and Shair, 2005). During the grief process, having a healthy, ongoing bond with the deceased can facilitate post-loss emotional processing, worldview reframing, and growth (Currier et al., 2015). Surprisingly, the impact of this change in the schema or internal representation of the mother was so powerful that it was even projected into the participant’s perception of the outside world as she looked at the photograph of her mother. To the best of our knowledge, the therapeutic potential of grief adaptation caused by the transformation of the internal working model of the deceased has not yet been specifically studied. This may be because the concept of internal working models is vague and untestable, and they are considered to be fixed, non-changing long-term representations. Future research is needed, focusing on equivalent and testable concepts such as cognitive schemas (Bosmans, 2009).

A similar process happened for the participant regarding the rest of her family, especially with her father, where the “conversation” with her mother helped her to reinterpret her family situation in a way that alters its meaning and changes its emotional impact. This cognitive reappraisal of the family grief processes led the client to regulate her emotions, transforming her anger into compassion. A follow-up would be necessary in this case to observe the long-term impact on the quality of family relationships.

Another aspect that emerged naturally during the ayahuasca experience is the resolution of “unfinished business.” “Unfinished business” is defined as any incomplete, unexpressed, or unresolved relationship issues with the deceased and has been considered a risk factor for chronic and severe grief reactions (Klingspon et al., 2015). Given that the participant longed to have an explanation of why her mother killed herself, the ayahuasca experience allowed her to understand that her mother was not able to cure herself in life. This information may not have been new for the participant, but it seemed important for her to receive it “directly” from her mother.

The elaboration of a narrative from the psychological content that emerged spontaneously, like love as the ultimate goal of existence, God as an “everything,” and the different levels of reality between life and death, implies the elaboration of superordinate or core features of a personal meaning system. These evoked superordinate constructs reflect central existential themes of meaning and purpose to a greater extent than subordinate constructs like personal or intellectual constructs (Neimeyer et al., 2001). Constructs functioning at this level of superordination are of fundamental importance for constructing the schemas of the self, others, and the world, which guide our behavior (Hinkle, 1965).

### Limitations

While this case report presents preliminary support for Restorative Retelling for processing psychedelic experiences, further validation in studies using larger samples is needed. Additionally, the lack of assessment after the ayahuasca experience and before Restorative Retelling procedure limits the differentiation of the potential contribution of the ayahuasca experience itself to the reduction of symptoms. Follow-up could determine whether changes observed during this procedure are stable for longer periods afterward. An additional limitation of our study is that this adaptation of the technique is composed of the assimilation and the accommodation processes, but the unique contribution of each could not be differentiated here. Only larger scale studies to assess each component will help to clarify this question. However, even after considering these limitations, this case report suggests that Restorative Retelling may be useful for processing and meaning-making of psychological content that emerges during psychedelic experiences.

## Conclusion

This study offers preliminary findings in support of Restorative Retelling as an effective technique for emotionally processing psychedelic experiences, fostering meaning-making in the life of the patients. Furthermore, this study shows how ayahuasca can evoke experiences that have a therapeutic potential in grief adjustment difficult to achieve with the classical therapeutic tools used in psychotherapy. Further research is needed to elucidate the therapeutic value of psychedelics by themselves from the contribution of the Restorative Retelling technique in the long-term relief of psychopathological symptoms and personal growth.

## Data Availability Statement

The original contributions presented in the study are included in the article/supplementary material, further inquiries can be directed to the corresponding author.

## Ethics Statement

The studies involving human participants were reviewed and approved by CEIC-Parc de Salut Mar, Barcelona, Spain. The patients/participants provided their written informed consent to participate in this study. Written informed consent was obtained from the individual(s) for the publication of any potentially identifiable images or data included in this article.

## Author Contributions

DG conceptualized the study design. DG and MBA collected the data and performed the intervention. JC analyzed the data. DG, RN, DN, and MF wrote the manuscript. All authors contributed to the article and approved the submitted version.

## Funding

This work was financially supported by the Riverstyx Foundation (United States) and the Verein Unfolding Light (Switzerland).

## Conflict of Interest

The authors declare that the research was conducted in the absence of any commercial or financial relationships that could be construed as a potential conflict of interest.

## Publisher’s Note

All claims expressed in this article are solely those of the authors and do not necessarily represent those of their affiliated organizations, or those of the publisher, the editors and the reviewers. Any product that may be evaluated in this article, or claim that may be made by its manufacturer, is not guaranteed or endorsed by the publisher.

## Footnotes

^1^ energycontrol-international.org

## References

[ref1] Alonso J. F. Romero S. Mañanas M. À. Riba J. (2015). Serotonergic psychedelics temporarily modify information transfer in humans. Int. J. Neuropsychopharmacol. 18, 1–9. doi: 10.1093/ijnp/pyv039, PMID: 25820842 PMC4571623

[ref3] Barbosa V. Sá M. Rocha J. C. (2014). Randomized controlled trial of a cognitive narrative intervention for complicated grief in widowhood. Aging Ment. Health 18, 354–362. doi: 10.1080/13607863.2013.833164, PMID: 24073815

[ref4] Bartholomew K. Horowitz L. M. (1991). Attachment styles among young adults: a test of a four-category model. J. Pers. Soc. Psychol. 61, 226–244. doi: 10.1037/0022-3514.61.2.226, PMID: 1920064

[ref5] Bauer S. Lambert M. J. Nielsen S. L. (2004). Clinical significance methods: a comparison of statistical techniques. J. Pers. Assess. 82, 60–70. doi: 10.1207/s15327752jpa8201_11, PMID: 14979835

[ref6] Boelen P. A. De Keijser J. Van Den Hout M. A. Van Den Bout J. (2007). Treatment of complicated grief: a comparison between cognitive-behavioral therapy and supportive counseling. J. Consult. Clin. Psychol. 75, 277–284. doi: 10.1037/0022-006X.75.2.277, PMID: 17469885

[ref7] Bosmans G. (2009). A cognitive perspective on attachment: The functioning of the internal working model and connections with the cognitive schema theory. Dissertation/Master’s thesis. Belgium: Ghent University.

[ref8] Bowlby J. (1982). Attachment and Loss. Vol. 1. Attachment. New York: Basic Books.

[ref9] Bruner J. (1987). Life as narrative. Soc. Res. 54, 11–32.

[ref10] Carhart-Harris R. L. Erritzoe D. Williams T. Stone J. M. Reed L. J. Colasanti A.. (2012). Neural correlates of the psychedelic state as determined by fMRI studies with psilocybin. Proc. Natl. Acad. Sci. U. S. A. 109, 2138–2143. doi: 10.1073/pnas.1119598109, PMID: 22308440 PMC3277566

[ref11] Chow A. Y. Chu K. S. (2016). “The dual rose” in Techniques of Grief Therapy: Assessment and Intervention. ed. Neimeyer R. A. (New York: Routledge), 259–263.

[ref12] Correa F. (2016). “Drawing images of violent death” in Techniques of Grief Therapy. ed. Neimeyer R. A. (New York: Routledge), 256–258.

[ref13] Currier J. M. Holland J. M. Neimeyer R. A. (2006). Sense-making, grief, and the experience of violent loss: toward a mediational model. Death Stud. 30, 403–428. doi: 10.1080/07481180600614351, PMID: 16610156

[ref14] Currier J. Irish J. Neimeyer R. Joshua F. (2015). Attachment, continuing bonds and complicated grief following violent loss: testing a moderated model. Death Stud. 39, 201–210. doi: 10.1080/07481187.2014.975869, PMID: 25551174

[ref15] Derogatis L.R. (1994). SCL-90-R. Symptom Checklist-90-R. Administration, Scoring and Procedures Manual. Minneapolis: National Computer System.

[ref17] Epstein S. (1990). “Cognitive-experiential self-theory” in Handbook of Personality Theory and Research: Theory and Research. ed. Pervin L. (NY: Guilford Publications), 165–192.

[ref18] Fiske S.T. (2004). Social Beings: A Core Motives Approach to Social Psychology. New York: Wiley.

[ref19] Foa E. B. Molnar C. Cashman L. (1995). Change in rape narratives during exposure therapy for posttraumatic stress disorder. J. Trauma. Stress. 8, 675–690. doi: 10.1007/BF02102894, PMID: 8564278

[ref20] Forstmann M. Yudkin D. A. Prosser A. M. Heller S. M. Crockett M. J. (2020). Transformative experience and social connectedness mediate the mood-enhancing effects of psychedelic use in naturalistic settings. Proc. Natl. Acad. Sci. U. S. A. 117, 2338–2346. doi: 10.1073/pnas.1918477117, PMID: 31964815 PMC7007572

[ref21] Furth H. G. (1969). Piaget and Knowledge: Theoretical Foundations. New Jersey: Prentice Hall.

[ref22] Garcia-Romeu A. Richards W. A. (2018). Current perspectives on psychedelic therapy: use of serotonergic hallucinogens in clinical interventions. Int. Rev. Psychiatry 30, 291–316. doi: 10.1080/09540261.2018.1486289, PMID: 30422079

[ref02] Gilbert K. R. (2002). Taking a narrative approach to grief research: Finding meaning in stories. Death Stud. 26, 223–239. doi: 10.1080/07481180211274 11973836

[ref23] González D. Aronovich A. A. Carvalho M. (2021). “The therapeutic use of ayahuasca in grief,” in Ayahuasca Healing and Science. eds. Labate B. C. Cavnar C. (Switzerland: Springer Nature), 209–226.

[ref24] González D. Cantillo J. Pérez I. Farré M. Feilding A. Obiols J. E.. (2020). Therapeutic potential of ayahuasca in grief: a prospective, observational study. Psychopharmacology 237, 1171–1182. doi: 10.1007/s00213-019-05446-2, PMID: 31938878 PMC7113212

[ref25] González D. Carvalho M. Cantillo J. Aixalà M. Farré M. (2019). Potential use of ayahuasca in grief therapy. Omega 79, 260–285. doi: 10.1177/0030222817710879, PMID: 28556759

[ref26] Gorman I. Nielson E. M. Molinar A. Cassidy K. Sabbagh J. (2021). Psychedelic harm reduction and integration: a transtheoretical model for clinical practice. Front. Psychol. 12:645246. doi: 10.3389/fpsyg.2021.645246, PMID: 33796055 PMC8008322

[ref03] Greenberg L. Angus L. (2004). “The contributions of emotionprocesses to narrative change in psychotherapy: a dialecticalconstructivist approach” in Handbook of narrative psychotherapy: Practice, theory, and research. eds. Angus L. McLeod J. (California: Thousand Oaks), 331–349.

[ref27] Greer G. R. Tolbert R. (1998). A method of conducting therapeutic sessions with MDMA. J. Psychoactive Drugs 30, 371–379. doi: 10.1080/02791072.1998.10399713, PMID: 9924843

[ref28] Grof S. (1994). LSD Psychotherapy. California: Hunter House.

[ref29] Hartogsohn I. (2017). Constructing drug effects: a history of set and setting. Drug Sci. Pol. Law 3:205032451668332. doi: 10.1177/2050324516683325

[ref30] Hartogsohn I. (2018). The meaning-enhancing properties of psychedelics and their mediator role in psychedelic therapy, spirituality, and creativity. Front. Neurosci. 12:129. doi: 10.3389/fnins.2018.00129, PMID: 29559884 PMC5845636

[ref31] Hayes J. Leudar I. (2016). Experiences of continued presence: on the practical consequences of ‘hallucinations’ in bereavement. Psychol. Psychother. 89, 194–210. doi: 10.1111/papt.12067, PMID: 26183119

[ref01] Hearn B. (2021). Psychedelics, mystical experiences, and meaning making: a renegotiation process with the challenges of existence. J. Humanist. Couns. 60, 180–196. doi: 10.1002/johc.12164

[ref32] Hedtke L. (2012). “Introducing the deceased” in Techniques of Grief Therapy: Creative Practices for Counseling the Bereaved. ed. Neimeyer R. A. (New York: Routledge), 253–255.

[ref33] Hinkle D.N. (1965). The change of personal constructs from the viewpoint of a theory of implications. Dissertation/Master’s thesis. Columbus: The Ohio State University.

[ref34] Irish M. Piguet O. (2013). The pivotal role of semantic memory in remembering the past and imagining the future. Front. Behav. Neurosci. 7:27. doi: 10.3389/fnbeh.2013.00027, PMID: 23565081 PMC3615221

[ref35] Jacobson N. S. Truax P. (1991). Clinical significance: a statistical approach to defining meaningful change in psychotherapy research. J. Consult. Clin. Psychol. 59, 12–19. doi: 10.1037/0022-006X.59.1.12, PMID: 2002127

[ref36] Jahn D. R. Spencer-Thomas S. (2018). A qualitative examination of continuing bonds through spiritual experiences in individuals bereaved by suicide. Religion 9, 1–15. doi: 10.3390/rel9080248

[ref37] Janoff-Bulman R. (2014). “Schema-change perspectives on posttraumatic growth,” in Handbook of Posttraumatic Growth. eds. Calhoun L. G. Tedeschi R. G. (NY: Routledge), 95–113.

[ref38] Jelinek L. Randjbar S. Seifert D. Kellner M. Moritz S. (2009). The organization of autobiographical and nonautobiographical memory in posttraumatic stress disorder (PTSD). J. Abnorm. Psychol. 118, 288–298. doi: 10.1037/a0015633, PMID: 19413404

[ref39] Klass D. Silverman R. Nickman S. L. (2014). Continuing Bonds: New Understandings of Grief. Washington DC: Taylor and Francis.

[ref40] Klingspon K. L. Holland J. M. Neimeyer R. A. Lichtenthal W. G. (2015). Unfinished business in bereavement. Death Stud. 39, 387–398. doi: 10.1080/07481187.2015.1029143, PMID: 26057117 PMC5093909

[ref41] Kosminsky S. P. Jordan R. (2016). Attachment-Informed Grief Therapy. New York: Routledge.

[ref42] Labate B.C. Araujo W.S. (2004). O Uso Ritual da Ayahuasca. Campinas: FAPESP/Mercado de Letras.

[ref43] Meikle S. E. Liknaitzky P. Rossell S. L. Ross M. Strauss N. Thomas N.. (2020). Psilocybin-assisted therapy for depression: how do we advance the field? Aust. N. Z. J. Psychiatry 54, 225–231. doi: 10.1177/0004867419888575, PMID: 31752499

[ref44] Metzner R. (2005). Sacred Vine of Spirits: Ayahuasca. Rochester: Park Street Press.

[ref45] Milman E. Neimeyer R. A. Fitzpatrick M. MacKinnon C. J. Muis K. R. Cohen S. R. (2017). Prolonged grief symptomatology following violent loss: the mediating role of meaning. Eur. J. Psychotraumatol. 8:1503522. doi: 10.1080/20008198.2018.1503522, PMID: 30128081 PMC6095024

[ref46] Milman E. Neimeyer R. A. Fitzpatrick M. MacKinnon C. J. Muis K. R. Cohen S. R. (2019). Prolonged grief and the disruption of meaning: establishing a mediation model. J. Couns. Psychol. 66, 714–725. doi: 10.1037/cou0000370, PMID: 31647284

[ref47] Mithoefer M. C. (2017). A Manual for MDMA-Assisted Psychotherapy in the Treatment of Posttraumatic Stress Disorder (Version 8). Available at: https://s3-us-west-1.amazonaws.com/mapscontent/research-archive/mdma/TreatmentManual_MDMAAssistedPsychotherapyVersion+8.1_22+Aug2017.pdf (Accessed November 15, 2021).

[ref48] Nayak S. Johnson M. W. (2021). Psychedelics and psychotherapy. Pharmacopsychiatry 54, 167–175. doi: 10.1055/a-1312-7297, PMID: 33285578

[ref49] Neimeyer R. A. (1999). Narrative strategies in grief therapy. J. Constr. Psychol. 12, 65–85. doi: 10.1080/107205399266226

[ref50] Neimeyer R. A. (Ed.) (2012a). “Retelling the narrative of the death,” in Techniques of Grief Therapy (New York: Routledge), 106–110.

[ref51] Neimeyer R.A. (2012b). Techniques of Grief Therapy. Creative Practices for Counseling the Bereaved. New York: Routledge.

[ref52] Neimeyer R. A. (2015). “Reconstructing meaning in bereavement,” in The Wiley Handbook of Personal Construct Psychology. eds. Winter D. A. Reed N. (Malden: Wiley Blackwell), 254–264.

[ref53] Neimeyer R.A. (2016). Techniques of Grief Therapy. Assessment and Intervention. New York: Routledge.

[ref54] Neimeyer R. A. (2019). Meaning reconstruction in bereavement: development of a research program. Death Stud. 43, 79–91. doi: 10.1080/07481187.2018.1456620, PMID: 30907718

[ref55] Neimeyer R. A. (2022). “Grief therapy as a quest for meaning,” in Handbook of Grief Therapies. eds. Steffen E. Milman E. Neimeyer R. A. (London: Sage).

[ref56] Neimeyer R. A. Anderson A. Stockton L. (2001). Snakes 843 versus ladders: a validation of laddering technique as a measure of hierarchical structure. J. Constr. Psychol. 14, 83–103. doi: 10.1080/10720530125990

[ref57] Neimeyer R. A. Rynearson E. K. (2022). “From retelling to reintegration: narrative fixation and the reconstruction of meaning,” in The Restorative Nature of Ongoing Connections With the Deceased: Exploring Presence Within Absence. eds. Burke L. A. Rynearson E. K. (New York: Routledge).

[ref58] Neimeyer R. A. Thompson B. E. (eds.) (2014). “Meaning making and the art of grief therapy” in Grief and the Expressive Arts: Practices for Creating Meaning (New York: Routledge), 3–13.

[ref59] Nelson K. L. Horowitz L. M. (2001). Narrative structure in recounted sad memories. Discourse Process. 31, 307–324. doi: 10.1207/S15326950dp31-3_5

[ref61] Osório F. D. L. Sanches R. F. Macedo L. R. Dos Santos R. G. Maia-de-Oliveira J. P. Wichert-Ana L.. (2015). Antidepressant effects of a single dose of ayahuasca in patients with recurrent depression: a preliminary report. Braz. J. Psychiatry 37, 13–20. doi: 10.1590/1516-4446-2014-1496, PMID: 25806551

[ref62] Pahnke W. N. Kurland A. A. Unger S. Savage C. Grof S. (1970). The experimental use of psychedelic (LSD) psychotherapy. JAMA 212, 1856–1863. doi: 10.1001/jama.1970.03170240060010, PMID: 5467681

[ref63] Palhano-Fontes F. Barreto D. Onias H. Andrade K. C. Novaes M. M. Pessoa J. A.. (2019). Rapid antidepressant effects of the psychedelic ayahuasca in treatment-resistant depression: a randomized placebo-controlled trial. Psychol. Med. 49, 655–663. doi: 10.1017/S0033291718001356, PMID: 29903051 PMC6378413

[ref64] Parro-Jiménez E. Morán N. Gesteira C. Sanz J. García-Vera M. P. (2021). Duelo complicado: Una revisión sistemática de la prevalencia, diagnóstico, factores de riesgo y de protección en población adulta de España. Ann. Psychol. 37, 189–201. doi: 10.6018/analesps

[ref65] Peri T. Gofman M. (2014). Narrative reconstruction: an integrative intervention module for intrusive symptoms in PTSD patients. Psychol. Trauma 6, 176–183. doi: 10.1037/a0031965

[ref66] Peri T. Hasson-Ohayon I. Garber S. Tuval-Mashiach R. Boelen P. A. (2016). Narrative reconstruction therapy for prolonged grief disorder—rationale and case study. Eur. J. Psychotraumatol. 7, 1–11. doi: 10.3402/ejpt.v7.30687, PMID: 27150596 PMC4858499

[ref67] Phelps J. (2019). “Training psychedelic therapists” in Advances in Psychedelic Medicine: State-of-the-Art Therapeutic Applications. eds. Winkelman M. J. Sessa B. (Santa Barbara, CA: Praeger), 274–291.

[ref68] Prado D. A. Pinto J. Crippa J. Santos A. Ribeiro S. Araujo D.. (2009). Effects of the Amazonian psychoactive plant beverage ayahuasca on prefrontal and limbic regions during a language task: a fMRI study. Eur. Neuropsychopharmacol. 19, 314–315. doi: 10.1016/S0924-977X(09)70469-9

[ref69] Preller K. H. Vollenweider F. X. (2016). Phenomenology, structure, and dynamic of psychedelic states. Curr. Top. Behav. Neurosci. 36, 221–256. doi: 10.1007/7854_2016_459, PMID: 28025814

[ref70] Prigerson H. G. Maciejewski P. K. Reynolds C. F. III Bierhals A. J. Newsom J. T. Fasiczka A.. (1995). Inventory of complicated grief: a scale to measure maladaptive symptoms of loss. Psychiatry Res. 59, 65–79. doi: 10.1016/0165-1781(95)02757-2, PMID: 8771222

[ref71] Rauch S. L. Shin L. M. Whalen P. J. Pitman R. K. (1998). Neuroimaging and the neuroanatomy of posttraumatic stress disorder. CNS Spectr. 3, 30–41. doi: 10.1017/S1092852900007306

[ref72] Riba J. Rodriguez-Fornells A. Urbano G. Morte A. Antonijoan R. Montero M.. (2001). Subjective effects and tolerability of the south American psychoactive beverage ayahuasca in healthy volunteers. Psychopharmacology 154, 85–95. doi: 10.1007/s002130000606, PMID: 11292011

[ref73] Riba J. Valle M. Urbano G. Yritia M. Morte A. Barbanoj M. J. (2003). Human pharmacology of ayahuasca: subjective and cardiovascular effects, monoamine metabolite excretion, and pharmacokinetics. J. Pharmacol. Exp. Ther. 306, 73–83. doi: 10.1124/jpet.103.049882, PMID: 12660312

[ref74] Rollo-Carlson C. (2015). “Letters to self” in Techniques of Grief Therapy. ed. Neimeyer R. A. (New York: Routledge), 190–194.

[ref05] Rynearson E. K. (2006). Violent death: Resilience and intervention beyond the crisis New York: Routledge.

[ref04] Salloum A. Rynearson E. K. (2006). “Family resilience after violent death” in Violent death: resilience and intervention beyond the crisis. ed. Rynearson E. K. (New York: Routledge), 47–63.

[ref76] Sánchez J. I. R. Rodríguez J. M. A. de la Peña Fernández M. E. (2002). SCL-90: Aplicación y análisis de sus propiedades psicométricas en una muestra de sujetos clínicos españoles. Psicopatol. Clín. Legal Forense 2, 1–19.

[ref77] Schultes R.E. Hofmann A. (1992). Plants of the Gods: Their Sacred, Healing, and Hallucinogenic Powers. Rochester: Healing Art Press.

[ref78] Shanon B. (2002). The Antipodes of the Mind: Charting the Phenomenology of the Ayahuasca Experience. Oxford: University Press on Demand.

[ref79] Shanon B. (2003). Altered states and the study of consciousness—the case of ayahuasca. J. Mind. Behav. 24, 125–153.

[ref80] Shear M. K. Boelen P. A. Neimeyer R. A. (2011). “Treating complicated grief converging approaches,” in Grief and Bereavement in Contemporary Society: Bridging Research and Practice. eds. Neimeyer R. A. Harris D. L. Winokuer H. R. Thornton G. F. (Routledge: Taylor & Francis Group), 139–162.

[ref81] Shear K. Frank E. Houck P. R. Reynolds C. F. (2005). Treatment of complicated grief: a randomized controlled trial. JAMA 293, 2601–2608. doi: 10.1001/jama.293.21.2601, PMID: 15928281 PMC5953417

[ref82] Shear K. Shair H. (2005). Attachment, loss, and complicated grief. Dev. Psychobiol. 47, 253–267. doi: 10.1002/dev.20091, PMID: 16252293

[ref83] Simon N. M. (2013). Treating complicated grief. JAMA 310, 416–423. doi: 10.1001/jama.2013.8614, PMID: 23917292 PMC4530627

[ref84] Steffen E. Coyle A. (2010). Can ‘sense of presence’ experiences in bereavement be conceptualized as spiritual phenomena? Ment. Health Relig. Cult. 13, 273–291. doi: 10.1080/13674670903357844

[ref85] Thomas G. Lucas P. Capler N. R. Tupper K. W. Martin G. (2013). Ayahuasca-assisted therapy for addiction: results from a preliminary observational study in Canada. Curr. Drug Abuse Rev. 6, 30–42. doi: 10.2174/15733998113099990003, PMID: 23627784

[ref86] Timmermann C. Kettner H. Letheby C. Roseman L. Rosas F. Carhart-Harris R. (2021). Psychedelics alter metaphysical beliefs. PsyArXiv. doi: 10.31234/osf.io/f6sjk [Preprint] PMC8611059 34815421

[ref87] Tomioka M. Shimura M. Hidaka M. Kubo C. (2008). The reliability and validity of a Japanese version of symptom checklist 90 revised. Biopsychosoc. Med. 2, 1–8. doi: 10.1186/1751-0759-2-19, PMID: 18957078 PMC2582234

[ref88] Wigren J. (1994). Narrative completion in the treatment of trauma. Psychol. Psychother. 31, 415–423. doi: 10.1037/0033-3204.31.3.415

[ref89] Winkelman M. J. (2017). The mechanisms of psychedelic visionary experiences: hypotheses from evolutionary psychology. Front. Neurosci. 11:539. doi: 10.3389/fnins.2017.00539, PMID: 29033783 PMC5625021

